# Tremor stability index: a new tool for differential diagnosis in tremor syndromes

**DOI:** 10.1093/brain/awx104

**Published:** 2017-07-01

**Authors:** Lazzaro di Biase, John-Stuart Brittain, Syed Ahmar Shah, David J. Pedrosa, Hayriye Cagnan, Alexandre Mathy, Chiung Chu Chen, Juan Francisco Martín-Rodríguez, Pablo Mir, Lars Timmerman, Petra Schwingenschuh, Kailash Bhatia, Vincenzo Di Lazzaro, Peter Brown

**Affiliations:** 1 Neurology Unit, Campus Bio-Medico University of Rome, Via Alvaro del Portillo 200, 00128, Rome, Italy; 2 Nuffield Department of Clinical Neurosciences, University of Oxford, Level 6, West Wing, John Radcliffe Hospital, OX3 9DU, Oxford, UK; 3 Medical Research Council Brain Network Dynamics Unit, Department of Pharmacology, University of Oxford, Mansfield Road, OX1 3TH, Oxford, UK; 4 Department of Neurology, University Hospital of Cologne, Kerpener Straße 62, 50924 Cologne, Germany; 5 Department of Neurology and Neuroscience Research Center, Chang Gung Memorial Hospital and University, Taipei, Taiwan; 6 Unidad de Trastornos del Movimiento, Servicio de Neurología y Neurofisiología Clínica, Instituto de Biomedicina de Sevilla (IBiS), Hospital Universitario Virgen del Rocío/CSIC/Universidad de Sevilla, Seville, Spain; 7 Centro de Investigación Biomédica en Red sobre Enfermedades Neurodegenerativas (CIBERNED), Spain; 8 Department of Neurology, University Hospital Marburg, Germany; 9 Department of Neurology, Medical University of Graz, Auenbruggerplatz 22, 8036 Graz, Austria; 10 Sobell Department of Motor Neuroscience and Movement Disorders, University College London, Queen Square, WC1N 3BG, London, UK

**Keywords:** tremor, Parkinson’s disease, clinical neurophysiology, movement disorders, neurophysiology

## Abstract

Misdiagnosis among tremor syndromes is common, and can impact on both clinical care and research. To date no validated neurophysiological technique is available that has proven to have good classification performance, and the diagnostic gold standard is the clinical evaluation made by a movement disorders expert. We present a robust new neurophysiological measure, the tremor stability index, which can discriminate Parkinson’s disease tremor and essential tremor with high diagnostic accuracy. The tremor stability index is derived from kinematic measurements of tremulous activity. It was assessed in a test cohort comprising 16 rest tremor recordings in tremor-dominant Parkinson’s disease and 20 postural tremor recordings in essential tremor, and validated on a second, independent cohort comprising a further 50 tremulous Parkinson’s disease and essential tremor recordings. Clinical diagnosis was used as gold standard. One hundred seconds of tremor recording were selected for analysis in each patient. The classification accuracy of the new index was assessed by binary logistic regression and by receiver operating characteristic analysis. The diagnostic performance was examined by calculating the sensitivity, specificity, accuracy, likelihood ratio positive, likelihood ratio negative, area under the receiver operating characteristic curve, and by cross-validation. Tremor stability index with a cut-off of 1.05 gave good classification performance for Parkinson’s disease tremor and essential tremor, in both test and validation datasets. Tremor stability index maximum sensitivity, specificity and accuracy were 95%, 95% and 92%, respectively. Receiver operating characteristic analysis showed an area under the curve of 0.916 (95% confidence interval 0.797–1.000) for the test dataset and a value of 0.855 (95% confidence interval 0.754–0.957) for the validation dataset. Classification accuracy proved independent of recording device and posture. The tremor stability index can aid in the differential diagnosis of the two most common tremor types. It has a high diagnostic accuracy, can be derived from short, cheap, widely available and non-invasive tremor recordings, and is independent of operator or postural context in its interpretation.

## Introduction

Misdiagnosis in tremor syndromes is a common and often underestimated problem that can cause misleading results in clinical trials (Rizzo *et al.*, 2016). At the clinical level misdiagnosis may lead to suboptimal treatment and incorrect prognosis. Central to this problem is the lack of accurate diagnostic tools that can distinguish different tremor aetiologies. Indeed, the diagnostic accuracy of Parkinson’s disease turns out to be only moderate when assessed against the gold standard of post-mortem histology (Gibb and Lees, 1988). Overall, diagnostic accuracy has been estimated to be 80% amongst movement disorders experts, and 74% if the disease is diagnosed by a neurologist not expert in movement disorders (Rizzo *et al.*, 2016). Thus, even using the UK Brain Bank criteria for Parkinson’s disease (Gibb and Lees, 1988) as a proxy for post-mortem examination, about 2 in 10 patients with Parkinson’s disease still receive a misdiagnosis, and this figure may be even higher in those presenting with Parkinson’s disease tremor (Selikhova *et al.*, 2013).

In essential tremor there is no gold standard diagnostic procedure, not even at post-mortem, and diagnosis is made purely on clinically-defined criteria (Deuschl *et al.*, 1998). Thirty-seven per cent of essential tremor patients are misdiagnosed, with the most common misdiagnosis being Parkinson’s disease tremor (Jain *et al.*, 2006). The differential diagnosis of essential tremor and Parkinson’s disease tremor is especially difficult early in the course of the disease when other parkinsonian signs may be absent and the clinician does not have the benefit of knowing the disease course (Deuschl *et al.*, 1998; Jain *et al.*, 2006; Bajaj *et al.*, 2010; Rizzo *et al.*, 2016). Moreover, patient age is not a discriminant factor, since early onset Parkinson’s disease and late onset essential tremor are part of the spectrum of these two diseases, and this often underlies the cases in which differential diagnosis is most difficult (Lou and Jankovic, 1991; Schrag and Schott, 2006).

Clinically, Parkinson’s disease tremor is present at rest, while tremor in essential tremor is postural and/or kinetic (Deuschl *et al.*, 1998; Shahed and Jankovic, 2007). However, Parkinson’s disease tremor may also manifest as a postural tremor, which generally appears a few seconds after assuming a posture—‘re-emergent’ tremor (Jankovic *et al.*, 1999; Shahed and Jankovic, 2007; Belvisi *et al.*, 2017). Nevertheless, this tremor onset delay can be absent in some patients with Parkinson’s disease, showing a pure postural tremor (Shahed and Jankovic, 2007). Conversely, if patients with essential tremor are not fully relaxed during the muscular tone evaluation, tremor can lead to the false impression of a cogwheel phenomenon (Elble, 2002; Shahed and Jankovic, 2007).

Given these uncertainties, ^123^I-FP-CIT and ^123^I-β-CIT DAT-SPECT have been used to help discriminate between Parkinson’s disease and essential tremor (Asenbaum *et al.*, 1998; Benamer *et al.*, 2000; Parkinson Study Group, 2000; Politis, 2014). However, the overall accuracy of nuclear imaging techniques for the Parkinson’s disease diagnosis may not be different from that of clinical diagnosis established by a movement disorder expert (de la Fuente-Fernández, 2012). Moreover, the use of this diagnostic support tool has created a new diagnostic grouping, defined as SWEDD (scans without evidence of dopaminergic deficit). The latter consists of patients that present clinically with parkinsonian features but do not have evidence of a dopaminergic deficit on presynaptic PET or SPECT (single-photon emission computed tomography) studies (Erro *et al.*, 2016). In clinical trials, the incidence of SWEDD is between 3.6 and 19.6% (Erro *et al.*, 2016). Although the most probable diagnosis underlying SWEDD may not be Parkinson’s disease, interpretation is confounded as some SWEDD patients do evolve to full-blown Parkinson’s disease (Menéndez-González *et al.*, 2014), and in the early stages of Parkinson’s disease a nuclear imaging deficit may not always be evident (Erro *et al.*, 2016). Meanwhile in essential tremor, there is insufficient evidence to support the use of nuclear imaging techniques for positive diagnosis (Colebatch *et al.*, 1990; Jenkins *et al.*, 1993; Wills *et al.*, 1994). Thus, nuclear imaging techniques can help distinguish tremor in Parkinson’s disease from that in other conditions, but are not perfect in this regard, and offer no help in distinguishing essential tremor from other non-parkinsonian forms of tremor such as that seen in dystonia. Finally, nuclear imaging techniques involve radiopharmaceutical agents, are expensive, time consuming, operator-dependent and not widely available.

In contrast, clinical neurophysiology is widely accessible, relatively inexpensive, and has also been explored as a diagnostic aid in tremor conditions (Deuschl *et al.*, 1996). However, with one possible exception, no neurophysiological techniques have proven to have good classification properties for Parkinson’s disease and essential tremor differential diagnosis, with confirmed validity in an independent cohort (Deuschl *et al.*, 1996). The latter helps establish the robustness of any metric to small variations in data recording procedures and patient demographics. The possible exception to this rule is the mean harmonic power (MHP) of postural tremor harmonics Muthuraman *et al.*, 2011; Wile *et al.*, 2014). This measure likely reflects differences in the structure of tremor EMG bursts between the two conditions. Nevertheless, its use has only been partially validated in two independent cohorts, as it proved necessary to have different cut-offs in the original and validation cohorts, perhaps because estimates of MHP rely on carefully calibrated accelerometer recordings (Muthuraman *et al.*, 2011; Wile *et al.*, 2014). The stability of tremor frequency over time has also recently been considered as the basis for a potential diagnostic aid. The instantaneous frequency of tremor and its temporal evolution is readily revealed by accelerometry—a cheap and simple means of recording tremor, and for this use devices do not require careful calibration across study populations. Brittain *et al.* (2015) analysed the variation in instantaneous tremor frequency over time, and showed that, in essential tremor, the frequency of tremor remains stable only over a narrow range of frequencies, whereas in Parkinson’s disease tremor the frequency can remain stable over a much broader range. These authors defined a new index, the frequency tolerance of tremor, as the frequency range over which tremor could settle at a temporarily stable frequency. However, the range of frequency tolerance was essentially established by considering the behaviour of tremor oscillations at outlying frequencies and as such did not capitalize on the whole tremor time series, nor characterize overall tremor stability.

Here we analyse the overall tremor stability characteristics of Parkinson’s disease and essential tremor, and use this information to develop a new measure, the tremor stability index (TSI), for the discrimination of these two tremor types. The utility and diagnostic performance of this index is analysed, and its performance validated in a separate patient cohort.

## Materials and methods

### Patients

All patients gave informed consent and the study was approved by local research ethics committees in accordance with the Declaration of Helsinki. In each dataset, tremor data were collected after overnight withdrawal of anti-parkinsonian and anti-tremor medications, and after at least 1 h of switching off any neurostimulation in patients implanted with deep brain stimulation (DBS) devices.

We considered two principal and independent cohorts, each drawn from more than one source, and collected with different devices (Table 1). This inhomogeneity served our aim to develop a diagnostic index that would be robust in the face of methodological differences and variation in demographics, and thereby reproducible and of practical utility. The inclusion criteria for patients were the clinical diagnosis of Parkinson’s disease or essential tremor made by experienced movement disorder specialists following the Queen Square Brain Bank diagnostic criteria for Parkinson’s disease patients (Hughes *et al.*, 1992) and the criteria of the Consensus statement of the Movement Disorder Society on Tremor (Deuschl *et al.*, 1998) for essential tremor patients. These clinical diagnoses served as the diagnostic gold standard against which neurophysiological measures were compared. The first ‘Test’ cohort comprised 16 rest tremor recordings of Parkinson’s disease patients from the University Campus Bio-Medico of Rome, Italy, and postural tremor recordings of 20 essential tremor patients from the University Hospital Cologne, Germany. The second, independent, ‘Validation’ cohort comprised both new data and original data drawn from previously published studies (Table 1), and afforded 42 rest tremor recordings of Parkinson’s disease patients and eight postural tremor recordings of essential tremor patients.

A third, ‘Postural context’ cohort was used to test whether the developed index discriminated between tremor types irrespective of postural context. To this end we collected another cohort with nine Parkinson’s disease patients who displayed re-emergent tremor and five Parkinson’s disease patients who had non-re-emergent postural tremor, and compared these with patients with essential tremor who had both tremor at rest and during posture taken from a previously published dataset in which clinical diagnosis was supported by SPECT-DaTSCAN imaging (Schwingenschuh *et al.*, 2010).

### Tremor recordings

Rest recordings were performed with the patient seated on a chair, with their forearms fully supported against gravity, and hands and wrists relaxed (Beuter *et al.*, 2001; Schwingenschuh *et al.*, 2010; Beudel *et al.*, 2015; Brittain *et al.*, 2015). Postural recordings were made, with the patient’s arms held outstretched against gravity in a horizontal, prone position (Schwingenschuh *et al.*, 2010). A triaxial accelerometer was used for tremor recordings, taped over the wrist in the test cohort. In the validation cohort, triaxial accelerometers were taped over the middle finger (*n* = 20) (Brittain *et al.*, 2015), thumb [*n* = 17 (Schwingenschuh *et al.*, 2010), and *n* = 5 (University of Seville)] or dorsal surface of the hand (*n* = 7) (Beudel *et al.*, 2015), or rest tremor recorded from the index finger using a velocity-transducing laser (*n* = 6) (Beuter *et al.*, 2001). In the postural context cohort, triaxial accelerometers were taped over the middle finger (*n* = 7) or the thumb (*n* = 17) (Schwingenschuh *et al.*, 2010). To compare the same amount of data among patients, the first 100 s of tremor was selected for analysis in each patient. The semiological characteristics of the tremor in each subject were determined from accelerometric recordings. To evaluate if the developed index could differentiate Parkinson’s disease from essential tremor, when estimated from EMG, we also derived this index from surface EMG recordings of wrist extensor and flexor muscles, in nine Parkinson’s disease and eight essential tremor patients (Schwingenschuh *et al.*, 2010).

### Data analysis

**Figure 1:**
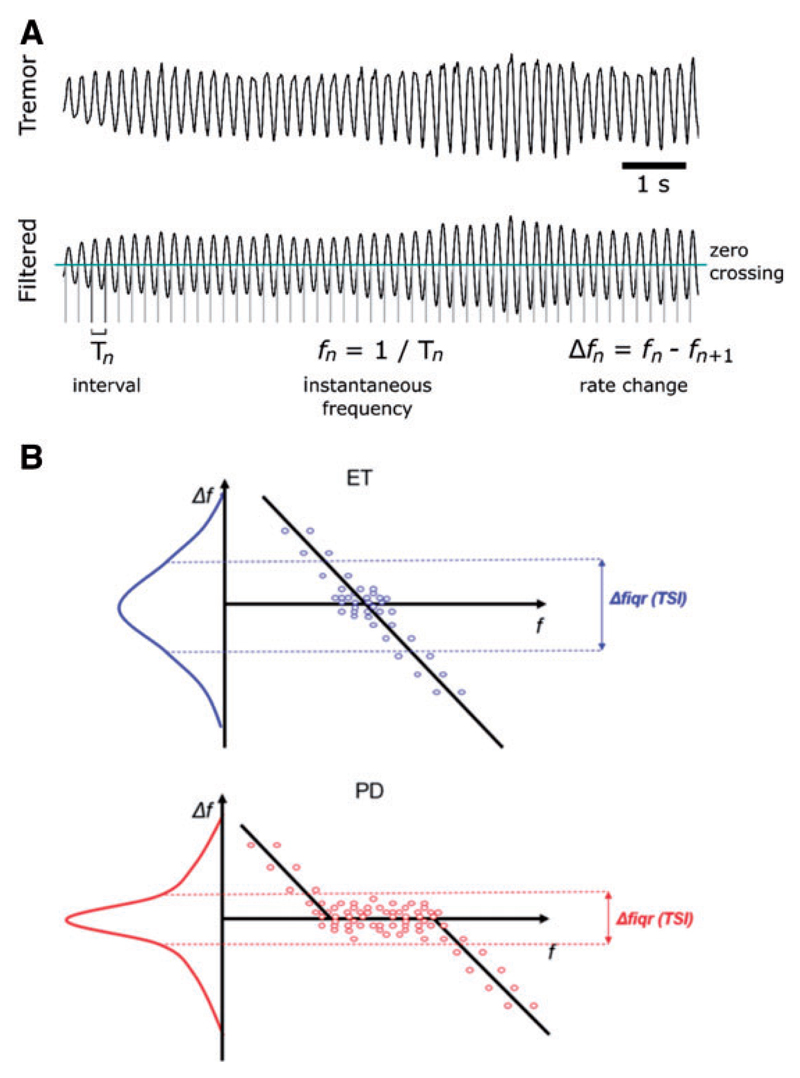
Schematic illustration of the procedure used to extract (A) the instantaneous frequency and variation in frequency, and (B) the TSI. The *lower* two graphs describe the relationship between instantaneous variation in frequency (∆*f*) and instantaneous frequency (*f*). Results are presented for an essential tremor (ET, *upper*) and Parkinson’s disease (PD, *lower*) patient. The essential tremor patient exhibits a linear ∆*f*/*f* relationship whereas the Parkinson’s disease patient displays a piecewise-linear relationship.

Data were converted from proprietary software to a universal format for analysis in Matlab (R2016a; Mathworks, USA). Triaxial accelerometer data were trend corrected [third-order zero-phase (forward-backward) high-pass Butterworth filter; 0.1 Hz corner frequency; *butter* and *filtfilt* routines in Matlab] and the dominant axis of tremor isolated by principal component analysis (PCA; *princomp* routine in Matlab). PCA is equivalent to a physical rotation of the sensor, thus ensuring that our analysis was primarily concerned with the orientation associated with the largest contribution to tremor irrespective of variations in sensor placement. The first principal component was chosen for further analysis. The peak frequency between 2–9 Hz was identified (denoted *f_c_*), and the data further filtered between (*f_c_–2*) and (*f_c_ + 2*) Hz using separate high- and low-pass filters (both zero-phase third-order Butterworth). The filtered data were then thresholded at zero and positive gradient crossings identified. Instantaneous frequencies (*f_n_*) were determined as the inverse of the interval between zero-crossings (*1*/*T_n_*), with tremor amplitude computed as the magnitude of the Hilbert envelope at each zero-crossing (*Hilbert* routine in Matlab, smoothed by 1 s). From the corresponding series of instantaneous frequencies *f_n_* we were able to calculate the cycle-by-cycle variation in tremor frequency, Δ*f* = *f_n_* − *f*_*n*+1_ (Brittain *et al.*, 2015) (Fig. 1). A measure of signal-to-noise ratio was constructed as 20·log_10_ of the (1 s smoothed) magnitude of the Hilbert envelope of the band-pass filtered signal, versus the ‘noise’ contribution, defined in this case as the Hilbert envelope of the unfiltered signal with the filtered signal subtracted. This definition does not account for the presence of harmonics in the data that may contribute to tremor. Once zero-crossings are defined our metrics of interest, such as the interquartile range of the change in frequency (Δ*f*), are readily computed. This new metric will be defined as TSI.

The latter can be partially related to the frequency tolerance reported by Brittain *et al.* (2015) (where the data were binned), but by design is an index that is estimated from the whole data distribution. To derive the frequency tolerance, instantaneous tremor frequency (*f*) is plotted against the expected change in tremor frequency on the next cycle (Δ*f*). This leads to a narrow range of tolerant frequencies in essential tremor and a broad range of frequency tolerance in Parkinson’s disease (Fig. 1B). In determining the TSI we plot the probability density function of Δ*f* (Fig. 1B), and describe numerically the variability in the Δ*f* distribution by extracting the interquartile range of Δ*f.*

The classification accuracy of TSI in the differential diagnosis of Parkinson’s disease and essential tremor was assessed by binary logistic regression using the ‘Enter’ method, and by receiver operating characteristic (ROC) analysis, using IBM SPSS Statistics (Zweig and Campbell, 1993). From SPSS, ‘B’ is the value, in log-odds units, for the logistic regression equation for predicting the dependent variable from the independent variable. By exponentiating the coefficient ‘B’, the resulting Exp(B) is an odds ratio, which indicates the change in odds resulting from a unit change in the predictor. The performance of TSI as a classifier was examined by calculating the sensitivity, specificity, accuracy, likelihood ratio positive, likelihood ratio negative and ROC area under the curve (AUC).

The TSI threshold for discriminating Parkinson’s disease and essential tremor was determined on the test dataset by selecting the cut-off, which maximized the distance between the true positive rate (sensitivity) and false positive rate (1 – specificity). This corresponds to the threshold with the highest combination of sensitivity and specificity values. Specific threshold values are inherently limited to those of the actual observations in the data. The threshold calculated from the test dataset was then cross-validated (10-fold) and verified in the second (validation) dataset, and in analysing the influence of postural dependencies on discriminability in the postural context dataset.

To evaluate the minimum recording duration needed for good classification performance, we applied a bootstrapping technique (1000 iterations) to the test dataset, by evaluating data lengths of 1 to 100 s (stepping in 1 s increments from 1–10 s and 10 s increments from 20–100 s). The bootstrapping technique also allowed us to estimate the variability of the distribution of diagnostic accuracy, measured with ROC AUC, by means of resampling different time lengths, each one extracted several times (1000 iterations), along the tremor recording time series for each patient.

To compute the MHP we first identified the peak tremor frequency from the spectral power of the first principal component of accelerometer data. All data were first transformed to units of milligravities [mg], then the peak spectral power was summed at its first four harmonic frequencies, $\text{MHP}=\text{log}\left(\frac{1}{4}\sum_{k=1}^{4}S(k\cdot f_{T})\right),$ with *f_T_* the peak frequency of tremor and *S*(*f*) the power spectrum (Muthuraman *et al.*, 2011). To compare the relative performance of MHP and TSI measures we bootstrapped the combined ‘Test and Validation’ dataset (10 000 iterations), computed both metrics and determined the difference in ROC AUC (TSI AUC minus MHP AUC) on a per iteration basis.

## Results

### Discriminating Parkinson’s disease and essential tremor

#### Test cohort

**Figure 2:**
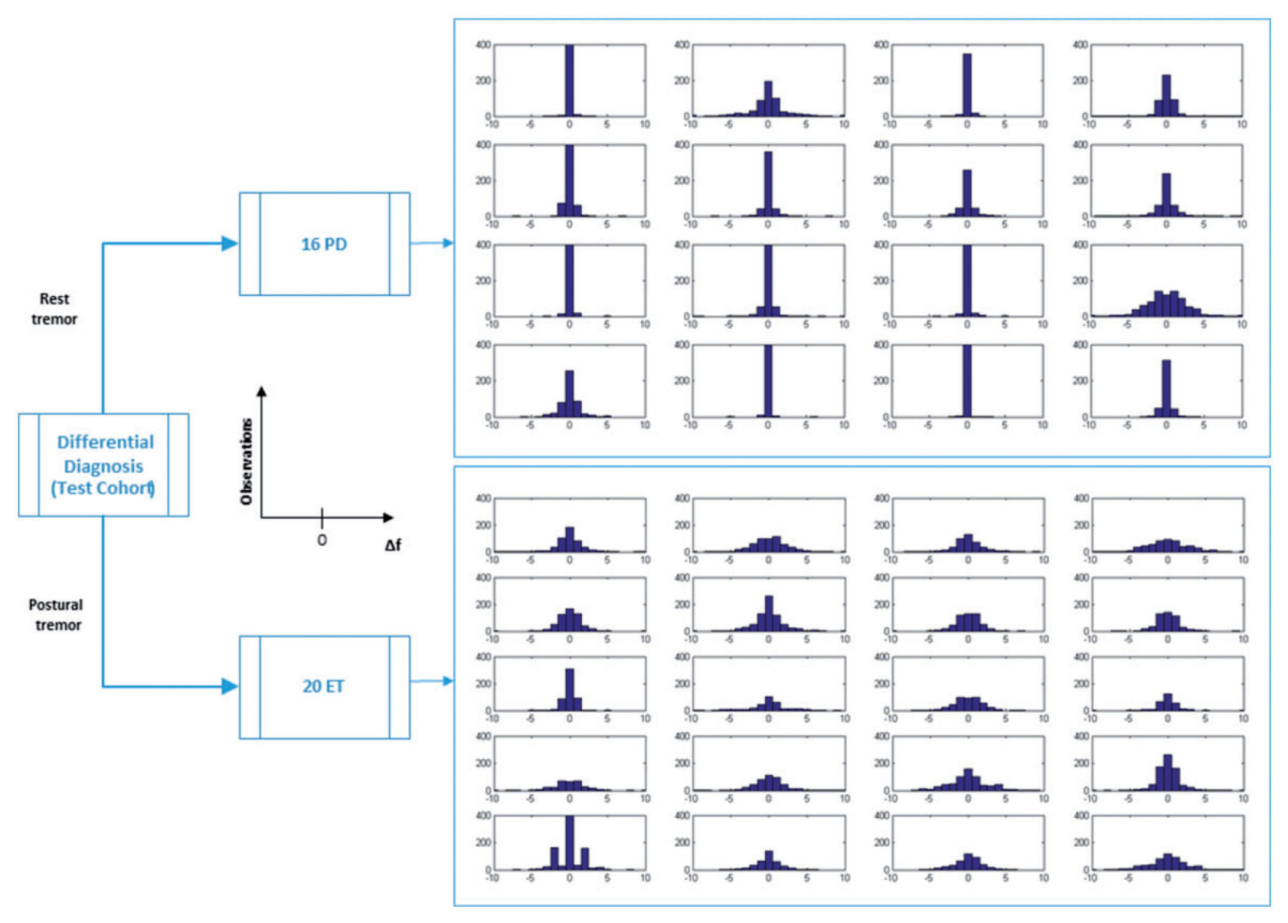
Test cohort: ∆f distribution. Instantaneous variations of the frequency (∆*f*) distribution are shown as histograms with the number of observations plotted against ∆*f* values for each of the Parkinson’s disease (PD) and essential tremor (ET) recordings in the test cohort. Parkinson’s disease tremor presents a narrower and sharper ∆*f* distribution than essential tremor.

We plotted the Δ*f* distribution for each patient in our test cohort in Fig. 2. Patients with Parkinson’s disease, in whom the tremor was recorded at rest, had a narrower and sharper Δ*f* distribution than patients with essential tremor, recorded whilst they maintained a tremor-provoking posture.

**Figure 3:**
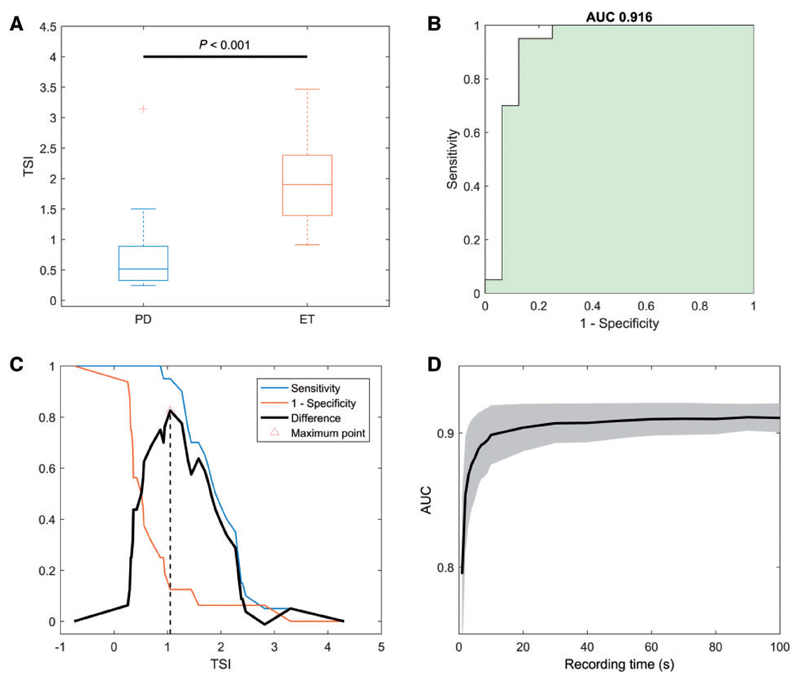
Test cohort: TSI diagnostic performance. **(A)** Boxplot comparing TSI distribution in Parkinson’s disease (PD) and essential tremor (ET) in the test cohort. T-test showed a significant difference between the two cohorts (*P* < 0.001). **(B)** ROC curve of the TSI as a diagnostic test differentiating Parkinson’s disease tremor from essential tremor, considering as target a diagnosis of essential tremor over Parkinson’s disease. AUC is 0.916 (95% CI 0.797–1.000), with a standard error of 0.06. **(C)** Plot of sensitivity and (1 – specificity) for each TSI value. The maximum distance between the sensitivity and 1 – specificity defines the highest combination of sensitivity and specificity values, and the corresponding best cut-off. **(D)** Recording duration boot-strapping on test dataset. Boot-strapping results for the ROC AUC values. Shaded regions are ± the standard deviation. All recording lengths longer than two or more seconds afforded better discrimination than chance, as determined by serial *t*-tests of the 19 different time lengths. One thousand iterations were performed per recording time.

To capture the difference in the distributions we calculated the Δ*f* interquartile range, hereafter termed the TSI, in each subject. Subjects were independently diagnosed through clinical evaluation made by one or more movement disorder experts. There was no difference of mean instantaneous frequency between patient groups [mean 5.08 ± 0.30 (SEM) Hz in Parkinson’s disease, 5.75 ± 0.28 Hz in essential tremor, *t*(34) = −1.652, *P* = 0.108]. There was a difference in TSI between groups [mean 0.7 ± 0.175 (SEM) in Parkinson’s disease, 1.9 ± 0.134 in essential tremor, *t*(34) = −5.481, *P* < 0.001; Fig 3A]. Binary logistic regression showed that for every unit increase in TSI, the odds [Exp(B)] of a patient having a diagnosis of essential tremor increased 14.8 times [95% confidence interval (CI) for Exp(B) 2.9–75.1; *P* = 0.001]. In addition, ROC analysis of TSI (considering as target a diagnosis of essential tremor over Parkinson’s disease) afforded an AUC of 0.916 (95% CI 0.797–1.000; Fig. 3B). TSI AUC outperformed the AUC value for mean frequency and measures of frequency variability (Table 2).

To find the optimal TSI threshold for differentiating essential tremor and Parkinson’s disease we selected the cut-off value of TSI, which maximized the distance between sensitivity and (1 – specificity). This approach maximizes combined sensitivity and specificity (Fig. 3C). The optimal TSI threshold in the test dataset was 1.05, so that TSI values > 1.05 were indicative of a diagnosis of essential tremor, and TSI values ≤ 1.05 indicative of Parkinson’s disease. The diagnostic performance of this TSI threshold was very good (Table 3).

Having determined the diagnostic potential of TSI we explored whether the TSI could be reliably estimated from clinically tractable tremor recording durations. The AUC of the ROC curve was 0.89 after as little as 10 s recording duration (Fig. 3D). This same analysis served to confirm that any 10-s period extracted from the original data performed in this way, i.e. the intraindividual variability of the TSI over time was limited.

To verify the stability of the TSI over time, we performed a follow-up of 10 Parkinson’s disease patients from the test cohort. The first recordings were made in July 2015, and the second recordings made 1 year and 7 months later. The follow-up recordings confirmed the stability of the TSI over time for Parkinson’s disease patients. There was no difference in TSI between the two recordings groups [mean 0.88 ± 0.279 (SEM) in Recording 1, 0.60 ± 0.093 in Recording 2, *t*(9) = 1.223, *P*=0.253]. The paired sample correlation for the two groups was 0.638, *P* < 0.05.

#### Validation cohort

**Figure 4:**
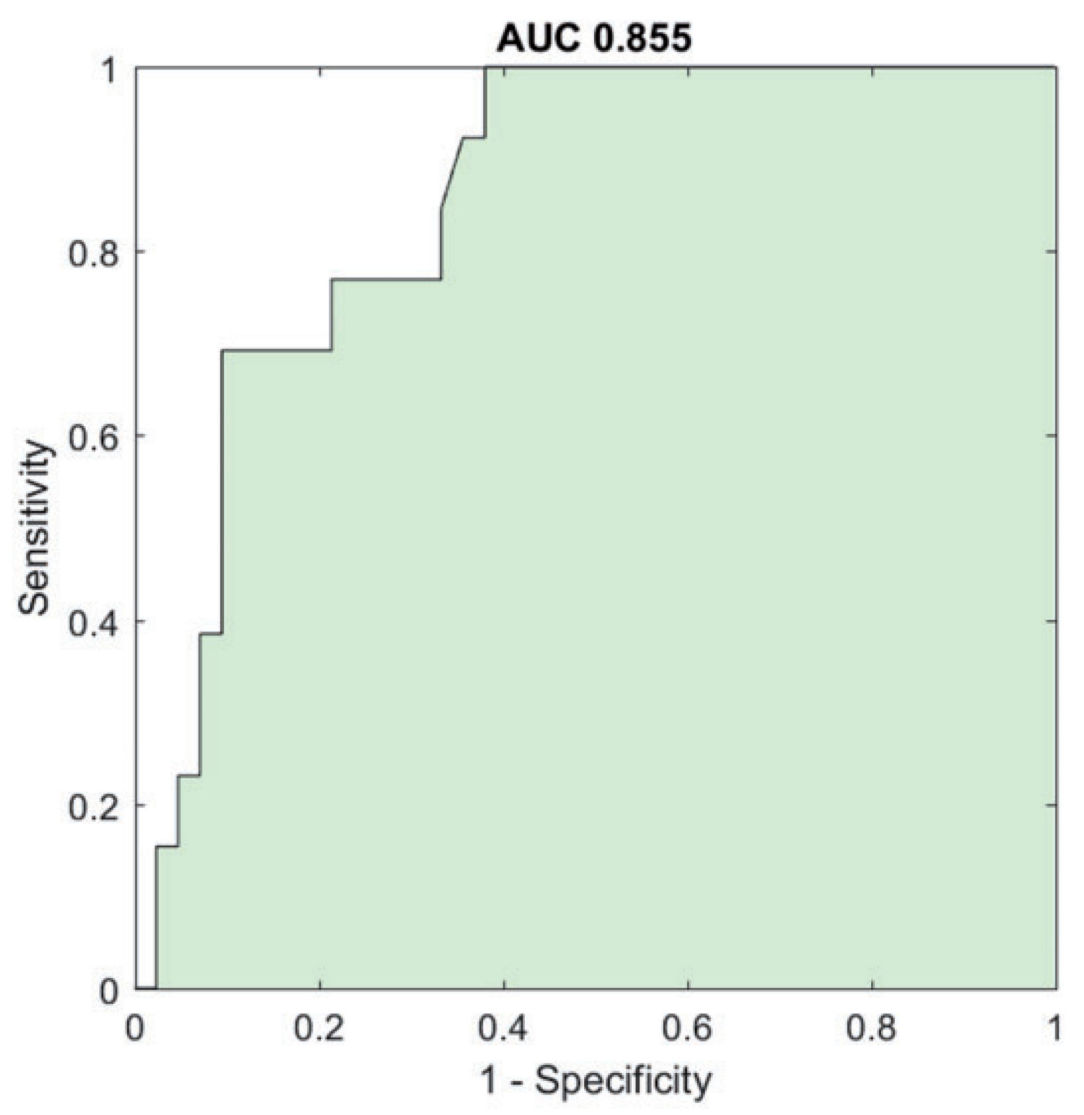
Validation cohort: ROC curve. ROC curve of the TSI as a diagnostic test applied for differential diagnosis of Parkinson’s disease and essential tremor in the independent validation cohort, considering as target a diagnosis essential tremor over Parkinson’s disease. AUC is 0.855 (95% CI 0.754–0.957) with a standard error of 0.052.

In the validation cohort, there was a difference of mean instantaneous frequency between groups [mean 4.98 ± 0.12 (SEM) Hz in Parkinson’s disease, 7.05 ± 0.27 Hz in essential tremor, *t*(53) = −7.763, *P* < 0.001]. A *t*-test confirmed a significant difference in TSI between essential tremor and Parkinson’s disease [TSI = 0.5 ± (SEM) 0.086 in Parkinson’s disease and 1.3 ± 0.194 in essential tremor; *t*(53) = −4.477; *P* < 0.001]. Binary logistic regression showed that for every unit increase in TSI, the odds [Exp(B)] of a patient having a diagnosis of essential tremor increased 5.7 times [95% CI for Exp(B) 2.0–15.7; *P* = 0.001]. In addition, ROC analysis of the TSI, considering as target a diagnosis of essential tremor over Parkinson’s disease, afforded an AUC of 0.855 (95% CI 0.754–0.957; Fig. 4).

Applying the same TSI threshold as for the test dataset we again found excellent diagnostic performance in discriminating essential tremor from Parkinson’s disease (Table 4). Despite the methodological differences and variation in demographics across the different datasets making up the validation cohort (Table 1), the diagnostic performance showed an excellent sensitivity for Parkinson’s disease diagnosis and specificity for essential tremor diagnosis.

The TSI data were compared to clinical diagnosis by one or more movement disorder experts in this study. However, the clinical diagnosis of tremor due to Parkinson’s disease was supported by SPECT-DaTSCAN imaging in one of the patient cohorts used for validation (Schwingenschuh *et al.*, 2010), and in this particular dataset a *t*-test confirmed a significant difference in TSI between essential tremor and Parkinson’s disease [TSI = 0.7 ± (SEM) 0.253 in Parkinson’s disease and 1.6 ± 0.229 in essential tremor; *t*(15) = −2.785; *P* = 0.014], and the diagnostic accuracy of the TSI was 82%.

### Comparison with state-of-the-art neurophysiology

The only electrophysiological measure so far identified for the differential diagnosis between Parkinson’s disease tremor and essential tremor with an accuracy >85% tested in an independent validation cohort is the MHP. We sought to assess the relative performance of TSI against MHP in our data. Across our combined dataset (test and validation cohorts) MHP produced a ROC AUC of 0.89 compared to 0.92 of TSI. Bootstrap analysis revealed that TSI outperformed MHP 72% of the time.

### Discriminating Parkinson’s disease and essential tremor: is the TSI independent of posture or recording device?

A further analysis, showed that TSI essential tremor versus Parkinson’s disease discrimination properties did not depend on whether tremor was recorded at rest or during postural contraction (Supplementary material). In addition, analysis showed that EMG could not replace kinematic sensors like accelerometers or lasers in the estimation of discriminative TSI (Supplementary material).

## Discussion

Misdiagnosis in Parkinson’s disease and essential tremor is common. We present a robust new objective measure, the TSI, which can be derived from short, simple and cheap recordings of tremor, and can discriminate Parkinson’s disease tremor and essential tremor with high sensitivity, specificity and diagnostic accuracy. The index’s utility was validated in a second, large and independent cohort, itself drawn from distinct patient datasets using different recording devices, and exhibiting demographic and clinical variation. The new index proved robust to such inhomogeneity, and moreover, its discriminative potential was not dependent on postural context. Regardless of the presence or absence of a statistically significant difference in mean instantaneous frequency between groups, the TSI consistently demonstrated good diagnostic performance in differentiating Parkinson’s disease from essential tremor patients.

The diagnostic accuracy of the TSI for essential tremor and Parkinson’s disease was ~90% across the different implementations with respect to clinical evaluation made by movement disorder experts. This compares with a diagnostic accuracy for early Parkinson’s disease of about 84% in radionucleotide studies (de la Fuente-Fernández, 2012), which themselves are costly and not widely available. Nevertheless, the clinical diagnosis of tremor due to Parkinson’s disease was supported by SPECT-DaTSCAN imaging in one of the patient cohorts used in the current study (Schwingenschuh *et al.*, 2010), and in this particular dataset TSI had an accuracy of 82%. Neurophysiological measures other than the TSI have been explored but generally afford only moderate discrimination of essential tremor and Parkinson’s disease at the single subject level, and have seldom been validated across different datasets (Deuschl *et al.*, 1996). The one possible exception to the above is MHP. MHP performed well in our cohort, but was consistently less accurate than TSI in discriminating between Parkinson’s disease and essential tremor.

The TSI can be rapidly estimated from only 10 s recording durations of tremor. Tremor recordings can be made with different accelerometer devices, with differing location on the hand, or with velocity-transducing laser techniques. Recordings are therefore non-invasive, and not kinematic sensor-dependent. Note, however, that EMG did not prove a basis for discriminative TSI estimation, perhaps because kinematic sensors capture the whole temporal variability of tremor, whereas EMG is more focal and only offers insight into the variability driven by sampled muscles. The TSI itself is quantitative and can be automatically and objectively estimated from tremor time series; and unlike radionucleotide studies it is not therefore operator-dependent. Furthermore, the interpretation of the TSI does not depend on whether tremor is recorded at rest or during postural contraction.

The TSI index provides a promising diagnostic aid in clinical practice, and could be a useful tool to avoid selection errors in clinical trials. It may also provide novel electrophysiological insights as it, like the related frequency tolerance measure, infers the behaviour of underlying central oscillator circuits from the peripheral tremor (Brittain *et al.*, 2015). Going forwards, it will be important to see if this index can also distinguish dystonic tremor, which may masquerade as Parkinson’s disease or essential tremor, and to establish whether or not the index and its discriminative potential, remains independent of pharmacological and other treatments. That said, the need to discriminate between tremor aetiologies usually precedes trials of treatment.

In conclusion, the TSI is a new neurophysiological measure that shows diagnostic validity at both the individual patient and population level, and which can aid differential diagnosis of the two most common tremor types—Parkinson’s disease and essential tremor. This new tool has a high diagnostic accuracy, and can be objectively derived from short, cheap, widely available and non-invasive tremor recordings.

## Supplementary material

Supplementary material is available at *Brain* online.

Supplementary material

## Figures and Tables

**Table 1:** Composition of cohorts

Cohort	Data source	Diagnosis	Number of tremor recordings	Recording state	Age (mean ± SD)	Disease duration (mean ± SD)	Sensor type - producer
**Test**	University Campus Bio-Medico of Rome	Parkinson's disease	16	Rest	66.4 ± 8.6	6.7 ± 7.7	Triaxial accelerometer (Opal sensor APDM, Inc)
	University Hospital Cologne, Germany	Essential tremor	20	Posture	49.5 ± 15.5	24.3 ± 13.9	Triaxial accelerometer (Brainvision acceleration sensor)
							
**Validation**	Previously published (Schwingenschuh *et al.,* 2010)^a^	Parkinson's disease	9	Rest	68.7 ± 8.2	6.5 ± 3.4	Triaxial accelerometer (Biometrics Ltd)
		Essential tremor	8	Posture	68.6 ± 7.8	20.8 ± 19.4	Triaxial accelerometer (Biometrics Ltd)
	Previously published (Brittain *et al.,* 2015)	Parkinson's disease	20	Rest	68.2 ± 8.4	12.5 ± 2	Triaxial accelerometer (Twente Medical Systems International, Biometrics Ltd)
	Previously published (Beuter *et al.,* 2001)^b^	Parkinson's disease	6	Rest	58.5 ± 9.8	11.5 ± 5.3	Velocity-transducing laser (Bruel and Kjaer)
	Previously published (Beudel *et al.,* 2015)^c^	Parkinson's disease	7	Rest	49.5 ± 9.5	12.5 ± 6.8	Triaxial accelerometer (Twente Medical Systems International)
	University of Seville	Essential tremor	5	Posture	51.4 ± 16.4	23.8 ± 16.4	Triaxial accelerometer EGAS3 (Entran Devices)
							
**Postural context**	University of Oxford	Parkinson's disease	4	Re-emergent	68.2 ± 7.8	10.0 ±8.0	Triaxial accelerometer (Twente Medical Systems International)
			3	Postural			
	Previously published (Schwingenschuh *et al.,* 2010)^a^	Parkinson's disease	5	Re-emergent and rest	68.7 ± 8.2	6.5 ± 3.4	Triaxial accelerometer (Biometrics Ltd)
			2	Postural and rest			
			2	Rest and low amplitude postural			
		Essential tremor	8	Posture and rest	68.6 ± 7.8	20.8 ± 19.4	Triaxial accelerometer (Biometrics Ltd)

a Clinical diagnoses supported by SPECT-DaTSCAN imaging. Parkinson’s disease patients in this group were also recorded during posture and essential tremor patients during rest. These data were used to supplement the third postural dataset.

b These patients underwent deep brain stimulation functional neurosurgery [neurosurgical targets: ventral intermedium nucleus (*n* = 2 patients), subthalamic nucleus (*n* = 3 patients), internal globus pallidus (*n* = 1 patient)].

c These patients underwent STN DBS.

SD = standard deviation.

**Table 2:** ROC AUC values of tremor neurophysiological parameters

	AUC^a^	Asymptotic 95% CI	
		Lower bound	Upper bound
TSI	0.916	0.797	1.000
Mean frequency	0.694	0.516	0.871
Δ*f*_SD_	0.784	0.612	0.957
Δ*f*_cov_	0.409	0.210	0.609
F_SD_	0.781	0.609	0.953
F_cov_	0.791	0.637	0.944

a ROC AUC considering as target a diagnosis of essential tremor over Parkinson’s disease. Δ*f*_SD_ = standard deviation of instantaneous variation of frequency; Δ*f*_cov_ = coefficient of variation of instantaneous variation of frequency; F_SD_ = frequency standard deviation; F_cov_ = frequency coefficient of variation.

**Table 3:** TSI diagnostic performance on test cohort

	Diagnosis	
	Essential tremor versus Parkinson’s disease	Parkinson’s disease versus essential tremor
Sensitivity	95%	88%
Specificity	88%	95%
Accuracy	92%	92%
Likelihood ratio positive	7.60	17.50
Likelihood ratio negative	0.06	0.13

**Table 4:** TSI diagnostic performance on validation cohort

	Diagnosis	
	Essential tremor versus Parkinson’s disease	Parkinson’s disease versus essential tremor
Sensitivity	69%	90%
Specificity	90%	69%
Accuracy	85%	85%
Likelihood ratio positive	7.27	2.94
Likelihood ratio negative	0.34	0.14

## Abbreviations

AUC: area under the curve
MHP: mean harmonic power of tremor
ROC: receiver operating characteristic
SWEDD: scans without evidence of dopaminergic deficit
TSI: Tremor Stability Index
