# Supplementary material for: Tremor stability index: a new tool for differential diagnosis in tremor syndromes
Source: Brain. Author manuscript; Available in PMC 2017 Jul 1. (PMC5493195; doi:10.1093/brain/awx104)
Supplement: Supplementary material [file NIHMS72707-supplement-Suppl.pdf]

Supplementary material

Discriminating Parkinson’s disease and essential tremor: Is the TSI independent of posture?

In the core analyses rest tremor in Parkinson’s disease was contrasted with postural tremor in essential tremor, so that differences might relate to the discrimination of the postural state rather than disease specific tremor characteristics. We therefore evaluated the TSI diagnostic accuracy in a third, *Postural context* cohort, in which, first we evaluated the TSI diagnostic accuracy in the differentiation of PD rest tremor and ET rest tremor, and then the TSI diagnostic accuracy in the differentiation of ET postural tremor from either PD postural re-emergent or non-re-emergent tremor. Of the 16 PD patients in the postural context cohort, 9 had classical re-emergent postural tremor, and 5 non-re-emergent postural tremor. An additional two patients had postural tremor that was of very low amplitude, and high frequency and could not necessarily be ascribed to PD, as opposed to, for example co-incident enhanced physiological tremor (Deuschl *et al.*, 1998). The postural tremor in these two cases was not further analyzed.

Parkinson’s disease rest vs essential tremor rest tremor

A t-test confirmed a significant difference in the TSI in essential tremor and Parkinson’s disease tremor present at rest (TSI = 0.7 ± (SEM) 0.175 in Parkinson’s disease and 2.5 ± 0.201 in essential tremor; $t(15)= -4.438$; $p < 0.001$). There was a difference of mean instantaneous frequency between groups (mean 5.06 ± 0.31 (SEM) Hz in Parkinson’s disease, 7.34 ± 0.33 Hz in essential tremor, $t(15)= -5.068$, $p < 0.001$). Binary logistic regression showed that for every unit increase in TSI, the odds (Exp(B)) of a patient having a diagnosis of essential tremor increased 8.8 times (95% C.I. for Exp(B) 1.4 – 56.9; $p = 0.022$). In addition, ROC curve analysis of the TSI, considering as target a diagnosis of essential tremor over Parkinson’s disease, afforded an AUC of 0.931 (95% C.I. 0.812 – 1.000) with a standard error of 0.06. We again applied the same TSI threshold as in the *Test* and *Validation* cohorts and found excellent diagnostic performance, despite all analysed tremor being recorded at rest (Table 1sup).

Table 1sup TSI diagnostic performance: postural context cohort, rest tremor.

	**Rest**	
	**Essential tremor vs Parkinson’s disease**	**Parkinson’s disease vs essential tremor**
**Sensitivity**	100%	78%
**Specificity**	78%	100%
**Accuracy**	88%	88%
**Likelihood ratio positive**	4.50	Inf*

**Likelihood ratio negative**	0.00	0.22

* Since the Likelihood ratio positive is sensitivity / (1 − specificity) the resulting Likelihood ratio positive is infinite.

Parkinson's disease re-emergent postural vs essential tremor postural tremor

We evaluated the TSI diagnostic accuracy in differentiating re-emergent Parkinson's disease tremor (n =9) from postural essential tremor (n = 8). There was a difference in mean instantaneous frequency between groups (mean 5.45 ± 0.21 (SEM) Hz in Parkinson's disease, 7.22 ± 0.35 Hz in essential tremor, t(15)= -4.437, p < 0.001). There was also a difference in the mean tremor onset delay between the groups (mean 3.9 ± 0.7s (SEM) in Parkinson's disease, 0.2 ± 0.1s in essential tremor, t(15)= 5.161, p < 0.001). A t-test confirmed a significant difference in postural tremor between the TSI in essential tremor and Parkinson's disease (TSI = 0.5 ± (SEM) 0.162 in Parkinson's disease and 1.6 ± 0.229 in essential tremor; *t*(15)= -4.296; p = 0.001). Binary logistic regression showed that for every unit increase in TSI, the odds (Exp(B)) of a patient having a diagnosis of essential tremor increased 19.8 times (95% C.I. for Exp(B) 1.7 – 236.3; p = 0.018). In addition, ROC curve analysis of the TSI, considering as target a diagnosis of essential tremor over Parkinson's disease, afforded an AUC of 0.931 (95% C.I. 0.812 – 1.000) with a standard error of 0.060. We again applied the same TSI threshold as in the other cohorts and found excellent diagnostic performance, despite all analysed tremor being recorded during posture (Table 2sup).

Parkinson's disease non-re-emergent postural vs essential tremor postural tremor

Finally, we evaluated the TSI diagnostic accuracy in differentiating non-re-emergent postural tremor in Parkinson's disease (n =5) from postural essential tremor (n = 8). There was a difference in mean instantaneous frequency between groups (mean 4.95 ± 0.44 (SEM) Hz in Parkinson's disease, 7.22 ± 0.35 Hz in essential tremor, t(11)= -4.014, p = 0.002). There was no difference in mean tremor onset delay between groups (mean 0.1 ± 0.1s (SEM) in non-re-emergent postural tremor in Parkinson's disease, 0.2 ± 0.1s in essential tremor, t(11)= -0.478, p = 0.642). A t-test confirmed a significant difference in the TSI in essential tremor and Parkinson's disease (TSI = 0.5 ± (SEM) 0.267 in Parkinson's disease and 1.6 ± 0.229 in essential tremor; *t*(11)= -3.222; p = 0.008). Binary logistic regression showed that for every unit increase in TSI, the odds (Exp(B)) of a patient having a diagnosis of essential tremor increased 13.3 times (95% C.I. for Exp(B) 1.1 – 158.1; p = 0.04). In addition, ROC curve analysis of the TSI, considering as target a diagnosis of essential tremor over Parkinson's disease, afforded an AUC of 0.925 (95% C.I. 0.766 – 1.000) with a standard error of 0.081. We again applied the same TSI threshold as in the other cohorts and found excellent diagnostic performance, despite all analysed tremor being recorded during posture (Table 2sup).

Table 2sup TSI diagnostic performance: postural context cohort, re-emergent and postural tremor.

	Parkinson’s disease re-emergent postural vs Essential tremor postural		Parkinson’s disease non-re-emergentpostural vs Essential tremor postural	
	**Essential tremor vs Parkinson’s disease**	**Parkinson’s disease vs essential tremor**	**Essential tremor vs Parkinson’s disease**	**Parkinson’s disease vs essential tremor**
**Sensitivity**	88%	89%	88%	80%
**Specificity**	89%	88%	80%	88%
**Accuracy**	88%	88%	85%	85%
**Likelihood ratio positive**	7.88	7.11	4.38	6.40
**Likelihood ratio negative**	0.14	0.13	0.16	0.23

Between Parkinson’s disease patients with re-emergent postural tremor and Parkinson’s disease patients with non-re-emergentpostural tremor, there was a difference in mean tremor onset delay (mean 3.9 ± 0.7s (SEM) for re-emergent tremor, 0.1 ± 0.1s for postural tremor, t(12)= 4.160, p = 0.001), but there was no statistically significant difference in mean TSI o mean instantaneous frequency between the two groups.

Can EMG replace kinematic recordings and maintain the discrimination of TSI?

We examined if EMG could replace kinematic sensor data and maintain good discrimination. Comparison of the TSI value extracted from the surface EMG activity of the wrist extensor and flexor muscles in a subgroup of patients with Parkinson’s disease (n =9) and (n= 8) essential tremor did not show any statistically significant difference (*t*(15)= -0.771; p = 0.453) between groups, with a mean TSI of 2.8 ± (SEM) 0.2 in Parkinson’s disease and of 3.1 ± 0.09 in essential tremor. This compares with the significant differences identified between Parkinson’s disease and essential tremor groups attained using accelerometry in the same subgroup of patients with Parkinson’s disease tremor and essential tremor (*t*(15)= -2.785; p = 0.014; mean TSI 0.6 ± (SEM) 0.175 in Parkinson’s disease and 1.6 ± 0.134 in essential tremor).

Potential Conflicts of Interest:

The authors declare that there are no conflicts of interest.
